# Targeting immunometabolism to treat COVID-19

**DOI:** 10.1093/immadv/ltab013

**Published:** 2021-06-02

**Authors:** Shane M O’Carroll, Luke A J O’Neill

**Affiliations:** School of Biochemistry and immunology, Trinity Biomedical Sciences Institute, Trinity College Dublin, Dublin, Ireland

**Keywords:** COVID-19, SARS-CoV-2, macrophage, immunometabolism, innate immune reprogramming

## Abstract

The COVID-19 crisis has emphasised the need for antiviral therapies to combat current and future viral zoonoses. Recent studies have shown that immune cells such as macrophages are the main contributors to the inflammatory response seen in the later inflammatory phase of COVID-19. Immune cells in the context of a viral infection such as SARS-CoV-2 undergo metabolic reprogramming to elicit these pro-inflammatory effector functions. The evidence of metabolic reprogramming in COVID-19 offers opportunities for metabolites with immunomodulatory properties to be investigated as potential therapies to combat this hyper-inflammatory response. Recent research indicates that the metabolite itaconate, previously known to be broadly antibacterial, may have both antiviral and immunomodulatory potential. Furthermore, low itaconate levels have shown to correlate with COVID-19 disease severity, potentially implicating its importance in the disease. The antiviral potential of itaconate has encouraged researchers to synthesise itaconate derivatives for antiviral screening, with some encouraging results. This review summarises the antiviral and immunomodulatory potential of immunometabolic modulators including metformin, peroxisome proliferator-activated receptor agonists and TEPP-46 as well as itaconate, and its derivatives and their potential use as broad spectrum anti-viral agents.

## Introduction

The innate immune system is our immune system’s first line of defence against viruses, such as SARS-CoV-2. It consists of a set of germline-encoded pathogen recognition receptors (PRRs) that function to recognise pathogen-associated molecular patterns. For viral recognition, these PRRs consist of toll-like receptors, RIG-I-like receptors, NOD-like receptors (NLRs), and DNA sensors. Each sense conserved molecular structures in viruses (largely nucleic acid-based) and induce intracellular signalling cascades that converge on transcription factor activation and subsequent transcription of genes encoding pro-inflammatory cytokines, chemokines, and type I interferons to regulate the immune response, induce inflammation and limit viral replication [1].

Apart from the well-known innate immune signalling triggered by a viral infection, metabolic change also occurs downstream of some of these sensors. This was first discovered in response to LPS from Gram-negative bacteria. LPS activation of toll-like receptor 4 pathway upregulates aerobic glycolysis and fatty acid synthesis with a concomitant reduction in mitochondrial respiration and β-oxidation in macrophages. Breakpoints occur in the tricarboxylic acid cycle (TCA) cycle after succinate and citrate, causing these metabolites to accumulate. The accumulation of citrate drives mitochondrial ROS (mtROS), nitric oxide accumulation, itaconate production and contributes to the pro-inflammatory phenotype of M1-like macrophages [2]. Citrate accumulation also causes acetyl-CoA to build up and contributes to epigenetic changes such as histone acetylation, facilitating transcription of inflammatory genes [3].

Accumulation of succinate drives Warburg-like metabolism and acts by inhibiting prolyl hydroxylases, thus increasing hypoxia-inducible factor 1-alpha (HIF1α) stabilisation. This drives glycolysis and also increases transcription of the pro-inflammatory cytokine interleukin-1β (IL-1β). Succinate accumulation also causes reverse electron transport (RET) causing mtROS production and the stabilisation of HIF1α [4, 5].

During viral infection the host undergoes two phases of response, the first phase is a pro-inflammatory viral limiting response and the second phase is the resolution and anti-inflammatory phase. Both phases are important to effective viral clearance and host recovery from infection, and mal-adaptive responses in either phase can result in pathologies not directly related to viral load or viral effects, but the immunopathology caused by cells of the immune system [6].

Given the importance of macrophages in both phases of the immune response and the demonstrated metabolic flexibility modulating macrophage phenotype this review focuses on the metabolic reprogramming and immunological response to metabolic modulation and metabolites as a potential therapeutic avenue to the treatment of the pathogenic inflammation associated with COVID-19.

## Metabolic reprogramming during viral infection

Much of the understanding of metabolic reprogramming in innate immunity stems from challenge with bacterial derived LPS in macrophages. However, there is an increasing appreciation for metabolic change in response to viral sensing. Metabolic reprogramming in host cells in response to viral infection facilitates an effective antiviral innate immune response. Many viruses, however, ‘hijack’ host cell metabolism in an immune evasion strategy. This is done by causing metabolic dysfunction, increasing flux through the pentose phosphate pathway (PPP) to increase nucleotide biosynthesis and diverting amino acid metabolism for virion replication, maturation, and viral dissemination [7]. Many viruses upregulate PPP by increasing aerobic glycolysis. Such experiments include influenza A infection of mammalian cell-lines, hepatitis C (HCV) infection of hepatocyte cell-lines, adenovirus, Kaposi’s sarcoma-associated herpesvirus infection of lymphatic endothelial cells, and Epstein-Barr virus infection of neural progenitor cells [7]. Viruses often upregulate glycolysis via viral proteins which interact with and regulate key enzymes. In hepatocyte cell-lines, Non-Structural Protein 5A (NS5A) from HCV binds and activates hexokinase, a rate-limiting glycolytic enzyme, to increase glycolytic rate [8]. Creating this Warburg-like metabolism also contributes to the production of type I interferons and inflammatory cytokines which aid in the anti-viral immune response [9].

Viral infection often activates HIF1α which contributes to anti-viral innate immunity by producing inflammatory mediators. However, HIF1α also upregulates glycolytic genes needed for virion replication and therefore benefits the virus [10]. Many viruses also modulate other metabolic pathways such as downregulating mitochondrial β-oxidation and upregulating fatty acid synthesis to aid virus envelopment for the release of the virus [11].

## Metabolic reprogramming in COVID19 contributes to the antiviral immune response

The progression from SARS-CoV-2 infection to severe COVID-19 is caused by a mal-adaptive immune response which is defined by failure to elicit a timely and robust type-I interferon innate immune response. This leads to increased viral load, followed by a hyperinflammatory immune response, such as acute phase reactants and inflammatory markers such as interleukin-6 (IL-6), tumour necrosis factor (TNF), IL1β, and interleukin-18 (IL-18) [12]. This bi-phasic nature of COVID-19 progression to severe disease, the early viral phase followed by the inflammatory phase, suggests that anti-inflammatory therapies would be suited to those who present later in the infection and have persistently increased inflammatory markers.

**Figure 1.**
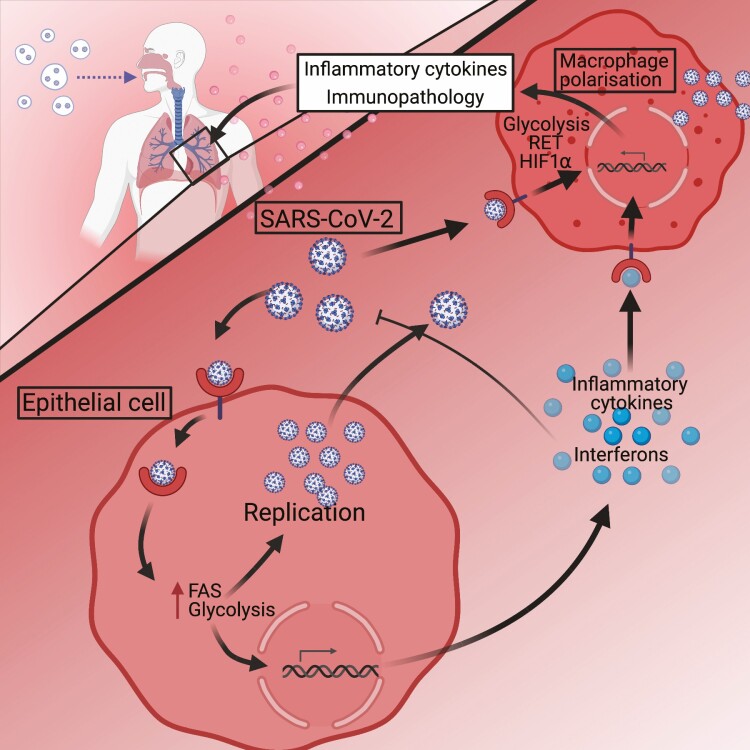
Schematic of SARS-CoV-2 infection in the lung. SARS-CoV-2 infection of airway epithelial cells and macrophages causes an upregulation of glycolysis and fatty acid synthesis (FAS), to enable viral replication and release of SARS-CoV-2. Viral sensing by PRRs causes interferon production, while increased glycolysis and FAS induces inflammatory cytokine production. Macrophages can be infected by SARS-CoV-2 in the same mechanism or can be polarised by inflammatory cytokines produced by airway epithelial cells. M1-like polarisation of macrophages causes the production of inflammatory cytokines that contribute to the immunopathology seen in COVID-19. Created with Biorender.com.

**Figure 2.**
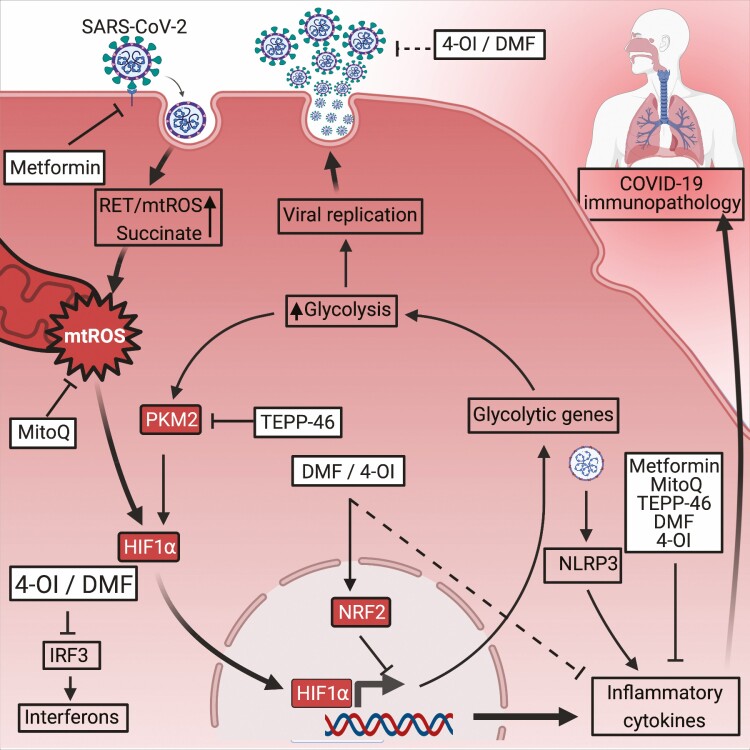
Metabolic modulators to treat COVID-19. 4-OI and DMF activate NRF2 which inhibit inflammatory cytokines. NRF2 activation prevents IRF-3 signalling, inhibiting interferon responses. 4-OI and DMF prevent TMPRSS2 expression which may prevent SARS-CoV-2 cell entry. MitoQ blocks SARS-CoV-2 replication. MitoQ increases autophagy and reduces mtROS to reduce inflammatory cytokines expression. TEPP-46 maintains PKM2 in an inactive conformation, preventing activation of HIF1α and associated inflammatory cytokines. Metformin phosphorylates ACE2 Ser680 which might prevent viral entry. Metformin upregulates ACE2, preventing RAS-mediated inflammation. Metformin inhibits inflammatory cytokine IL1β and upregulates anti-inflammatory cytokine IL-10. Created with Biorender.com.

Monocytes and macrophages are the most abundant immune cell-types in SARS-CoV-2-infected lungs and contribute to hyper-inflammatory cytokine production evident in severe COVID-19 [13]. SARS-CoV-2 infects many cell types including airway epithelial cells and macrophages. Infection of macrophages causes metabolic reprogramming, similar to that created by LPS, causing an increase in aerobic glycolysis, reduction of the TCA cycle and RET (See Figure 1) [14]. This metabolic reprogramming is mediated by the production of mtROS, stabilisation of HIF1α and HIF1α-mediated transcription of core glycolytic genes involved in glucose import and glycolysis including Glucose transporter 1 (GLUT-1), Pyruvate kinase M2 (PKM2), Lactate dehydrogenase A (LDH-A), and 6-Phosphofructo-2-Kinase/Fructose-2,6-Biphosphatase 3 (PFKFB3). It has also been shown that lactate dehydrogenase, an important enzyme in Warburg metabolism in M1-like macrophages, is a prognostic marker of disease severity in COVID-19 and further highlights the role of glycolysis in COVID-19 [15]. *Ex-vivo* experiments using human monocytes from SARS-CoV-2-infected patients showed that HIF1α protein was significantly increased when compared to uninfected patients. *In-vitro* experiments with SARS-CoV-2-infected human monocytes elegantly demonstrate that elevated glucose levels and glycolysis facilitate SARS-CoV-2 replication, as blocking glycolysis inhibits viral replication. Furthermore, the increased glycolysis also increases the production of IL-1β which contributes to the hyperinflammatory response of COVID-19. These *in-vitro* experiments directly implicate HIF1α in viral replication and inflammatory cytokine production as HIF1α inhibition was shown to inhibit SARS-CoV-2 replication and HIF1α activation increased viral replication. Furthermore, HIF1α inhibition also blocked transcription of inflammatory cytokines associated with severe COVID19 including IL1β, TNF, IL6, interferon alpha (IFNα), interferon beta (IFNβ), and Angiotensin-converting enzyme 2 (ACE2) [14]. This makes compelling evidence for targeting mtROS-HIFα-metabolic reprogramming as a potential treatment of severe COVID-19 by inhibiting viral replication and the inflammatory cytokines seen in COVID-19 pathology (see Figure 2) [14].

## Itaconate and COVID-19

Given these metabolic features, and the discovery that low plasma itaconate levels correlate with COVID-19 disease severity, it seems likely that itaconate might play a role in COVID-19 [16]. Itaconate is a metabolite produced by the decarboxylation of the TCA cycle intermediate cis-aconitate, a reaction catalysed by aconitate decarboxylase 1 (ACOD1), also referred to as immune responsive gene 1 (IRG1) [17, 18]. IRG1 and itaconate have been closely associated with the metabolic reprogramming of macrophages *in-vitro* into an M1-like phenotype as IRG1 is one of the most upregulated genes in classically activated macrophages [19]. The main role of itaconate in macrophages is to act in a negative feedback fashion, limiting inflammation [20]. Notably, itaconate is also antibacterial against many pathogens including *Salmonella enterica* and *Mycobacterium tuberculosis* (Mtb). These bacteria use carbon sources produced by the glyoxylate shunt to survive during human infection. Itaconate inhibits this glyoxylate shunt via blocking a key enzyme isocitrate lyase. Itaconate also stops Mtb from using carbon sources from cholesterol degradation by inhibiting Mtb mediated detoxification of propionic acid [21].

As well as being anti-bacterial, itaconate is also immunomodulatory. Biochemically, itaconate has been shown to alkylate cystine residues, resulting in changes to protein structure and binding. *In-vitro* experiments have shown that exogenous itaconate treatment of bone-marrow-derived macrophages upregulates IFN-β in a yet undetermined mechanism [22]. Also, itaconate and its derivative 4-Octyl itaconate, (4-OI), inhibits NOD-, LRR-, and pyrin domain-containing protein 3 (NLRP3) inflammasome activation, preventing the secretion of IL-1β [23] which is implicated in COVID-19 immunopathology [24]. Furthermore, itaconate reduces other inflammatory cytokines such as IL-6 and interleukin-10 (IL-10) [22, 25].

Not only has itaconate potential immunomodulatory effects, but itaconate has previously been shown to be antiviral against Zika virus (ZIKV) in murine models (see Table 1). ZIKV infection of murine neurons is sensed by the Z-form nucleic acid sensor (ZBP1). ZBP1 causes necroptotic-independent signalling via receptor-interacting protein kinase-1 (RIPK1) and receptor-interacting protein kinase-2 (RIPK2) leading to Interferon Regulatory Factor 1(IRF1)-dependant upregulation of IRG1 and concomitant itaconate synthesis. In this case, itaconate inhibits succinate dehydrogenase, causing succinate accumulation and driving an antiviral metabolic state in neurons via reduced TCA cycle metabolism and reduced oxygen consumption rate [26]. This effect was emphasised by demonstrating that IRG1 knock-out mice have higher levels of ZIKV replication [26]. Furthermore, IRG1 mediates an antiviral state in primary cortical neurons against West Nile Virus (WNV), and subsequent knockdown of IRG1 enhanced WNV infection [27]. This suggests that itaconate could have antiviral properties against ZIKV, WNV and possibly SARS-CoV-2 as they are all positive-strand RNA viruses (see Table 1).

**Table 1.** Antiviral immunomodulators

	SARS-CoV-2	WNV	ZIKV	HSV1/2	VACV	Influenza A
Itaconate	ND	(-)	(-)	ND	ND	(-)
4-OI	(-)	ND	(-)	(-)	(-)	ND
DMF	(-)	ND	(-)	(-)	(-)	ND
Metformin	(-)	ND	(-)	ND	ND	(-)

Viral inhibition is annotated with ‘(-)’, not determined is annotated with ‘ND’.

An interesting link from itaconate to SARS-CoV-2 was recently reported. 4-OI, a derivative of itaconate, has been shown to activate nuclear factor-erythroid factor 2-related factor 2 (NRF2) via the alkylation of Kelch Like ECH Associated Protein 1 (KEAP1) [28]. Furthermore, NRF2 activation is impaired in IRG-1-deficient macrophages treated with LPS. Of note, itaconate can restore LPS-induced NRF2 activation in IRG-1-deficient macrophages [29, 30]. This is interesting as NRF2 functions as a transcription factor that binds to the promoter region of pro-inflammatory genes, preventing RNA Polymerase II binding and also inhibits the transcription of pro-inflammatory cytokines IL6 and IL1β in macrophages [31]. NRF2 also drives antioxidant genes which reduce mtROS and HIF1α-mediated inflammation. Transcriptome analysis of severe COVID-19 patients has shown that NRF2 dependant genes are suppressed including Heme Oxygenase 1 (HMOX1) and NAD(P)H Quinone Dehydrogenase 1 (NqO1) [32]. This suggests that these NRF2 activators could be used as therapies for COVID-19.

Both 4-OI and dimethyl fumarate (DMF) are both NRF2 activators and inhibit SARS-Cov-2 replication in human airway epithelial cells. This antiviral effect also extends to Zika virus, Herpes Simplex Virus-1 (HSV1), Herpes Simplex Virus-2 (HSV2), and Vaccinia virus (VACV) (see Table 1). Furthermore, 4-OI and DMF inhibit the pathogenic interferon response in an NRF2-dependant mechanism by blocking interferon regulatory factor 3 (IRF-3) dimerisation. This was shown by demonstrating that silencing NRF2 rescues IRF-3 signalling. Additionally, NRF2 also reduces interferon responses by reducing stimulator of interferon genes (STING) expression [33]. Interestingly, 4-OI and DMF also inhibit SARS-CoV-2 replication in epithelial cells and human peripheral blood mononuclear cells (PBMCs) in a yet undetermined NRF2-independent mechanism.

Importantly, it was also shown that 4-OI and DMF blocked the expression of inflammatory factors including IFNβ1, C-X-C motif chemokine ligand 10 (CXCL-10), TNF, IL-1β, and C-C Motif Chemokine Ligand 5 (CCL5) induced by SARS-CoV-2 infection. Furthermore, pre-treatment with 4-OI or DMF upregulate anti-inflammatory markers such as HMOX1, indicating an increase in the anti-inflammatory responses. This anti-inflammatory effect was also seen in *ex vivo* PBMC samples of severe COVID-19 patients treated with 4-OI. These results show that 4-OI and DMF inhibit SARS-CoV-2 replication in human airway epithelial cells, while also suppressing the macrophage-mediated pathogenic interferon and inflammatory responses seen in the later inflammatory phase of COVID-19 (see Figure 2) [32].

Other Itaconate derivatives are directly antiviral against Influenza A Virus by binding to the viral nucleoprotein and blocking ribonucleoprotein export from the nucleus to the cytosol and preventing the replication cycle of the virus [34].

## Other metabolic targets in SARS-CoV-2 infection

Mitochondrial dysfunction and the production of mtROS are common features of SARS-CoV-2 infection in human macrophages *ex-vivo* and *in vivo* and causes the stabilisation of HIF1α and the subsequent production of pro-inflammatory cytokines [14]. This mechanism has also been seen in SARS-CoV-1 studies demonstrating that nucleocapsid protein N disrupts mitochondrial membrane potential and increases mtROS *in vitro* [35]. Furthermore, SARS-CoV-2 infection of bronchial epithelial cells reduces mitochondrial gene expression, leading to mitochondrial dysfunction and increasing mtROS *in vitro* [36]. Proteins involved in mitochondrial fission are also suppressed, increasing mitochondrial fusion and which impairs antiviral interferon responses [37].

With this in mind, MitoQ is a mitochondria-specific antioxidant that increases autophagy and reduces mtROS *in vitro* and in murine models [38]. Additionaly, studies demonstrate that MitoQ has antiviral potential. *In vivo* mouse models have shown that MitoQ reduce dextran sulfate sodium-induced colitis by reducing inflammatory cytokines IL-1β, TNF, and mtROS [39, 40]. Furthermore, *in-vitro* models of Respiratory syncytial virus (RSV) infection of vero and HEK293T-cells have shown that MitoQ suppresses RSV viral infection by blocking mitochondrial dysfunction and mtROS production [39, 41]. Importantly, an *in-vitro* study using SARS-CoV-2 infection of human monocytes shows that MitoQ blocks SARS-CoV-2 replication, and reduces HIF1α stabilisation and inflammatory cytokine IL-1β and ACE2 (see Figure 2) [14]

Increased glycolysis is also associated with SARS-CoV-2 replication. PKM2 is an enzyme that catalyses the final step of glycolysis and also has important roles in immune cell reprogramming and inflammation. However, PKM2 activity depends on its oligomerisation state. PKM2 tetramers are inactive, whereas phosphorylation of PKM2 forms a dimer that enters the nucleus, binds HIF1α, and causes the expression of proinflammatory and glycolytic genes [42]. In the context of COVID-19, both PKM2 levels and phosphorylated PKM2 levels increase with COVID-19 severity. This indicates that PKM2 is important in COVID-19 severity via activation of HIF1α and the expression of inflammatory cytokines [43]. This metabolic reprogramming causes cytosolic succinate accumulation which activates HIF1α. This is supported by *ex-vivo* COVID-19 studies show that succinate levels and nuclear translocation of both HIF1α and PKM2 positively correlate with COVID-19 disease severity [43]. Maintaining PKM2 as a tetramer could therefore be a potential therapy for COVID-19. Compounds such as TEPP-46 maintain PKM2 in a tetramer, inhibiting Warburg-like metabolism, succinate accumulation, inflammatory cytokine IL-1β and inhibits HIF1α dependant transcription of glycolytic genes needed for SARS-CoV-2 (see Figure 2) [44].

Other potential immunomodulators being proposed for the treatment of COVID-19 include peroxisome proliferator activated receptor (PPAR) agonists. PPARs are a group of transcription factors which regulate glucose and lipid metabolism as well as macrophage polarisation [45]. These transcription factor agonists may be a therapy to dampen the late inflammatory phase of COVID-19 as PPAR-γ agonists such as rosiglitazone can block nuclear factor kappa B (NF-κB)-mediated inflammatory cytokine production in lung epithelial cells [46, 47]. In the case of COVID-19, lung biopsies of COVID-19 patients show PPAR-γ is suppressed in M1-like macrophages, similar to that of PPAR-γ knockout macrophages. Furthermore SUMO1, a protein in the PPAR-γ complex was shown to be repressed in severe COVID-19 and shown to interact with a SARS nucleoprotein [48]. This suggests that SARS-CoV-2 may interact with SUMO1, repress PPAR-γ activity, leading to the hyperinflammatory response seen in severe COVID-19. PPAR-γ agonists could, in theory, be suggested as a therapy to dampen the excessive inflammation seen in the lungs of COVID-19 patients.

## Metformin and DMF in COVID-19

Metformin is a safe, widely used glucose lowering agent used to treat type 2 diabetes mellitus (T2DM). The anti-viral potential of metformin was first discovered in the 1940s when it showed significant anti-influenza properties [49]. Metformin has been shown to block ZIKA virus replication, increase innate immune signalling via interferon-stimulated genes such as interferon stimulated gene 15 (ISG15), 2′-5′-Oligoadenylate Synthetase 2 (OAS2) and blocks TNF and CCL5 production *in vitro* and *in vivo* (see Table 1) [50].

Metformin significantly reduced hospital mortality in diabetic patients with COVID-19, suggesting therapeutic potential [51]. Metformin’s anti-viral effect could be attributed to several potential mechanisms. SARS-CoV-2 entry into cells causes the downregulation of ACE2. ACE2 downregulation dysregulates the rennin-angiotensin system (RAS) which may contribute to increased inflammation. Metformin induces ACE2 expression in Human umbilical vein endothelial cells, potentially preventing RAS dysregulation and reducing inflammation [52]. Furthermore, in human endothelial cells, metformin causes increased phosphorylation of ACE2 Ser680 via AMPK. This might suggest that ACE2 phosphorylation might block spike-ACE2 interaction or binding and therefore reduce SARS-CoV-2 cell entry (see Figure 2) [53]. Metformin also blocks IL1β production and increases the anti-inflammatory cytokine IL-10 and inhibits complex 1 in mitochondria, indicating its anti-inflammatory potential for COVID-19 [40]. Furthermore, neutrophil extracellular traps (NETs) contribute to the immunopathology seen in COVID-19 [54]. Metformin inhibits NETs in diabetic patients [55]. Metformin has also been shown to block mechanistic target of rapamycin (mTOR). Sirolimus, another mTOR inhibitor has been shown to block MERS-CoV infection *in vitro* and significantly improve ICU patient outcome [56, 57]. Clinical trials investigating mTOR inhibitors against COVID-19 are currently recruiting participants. Metformin might have potential as a therapy for COVID-19.

Dimethyl fumarate (DMF) is a common therapy for adult patients with relapsing-remitting multiple sclerosis [58, 59]. Studies suggest that DMF might have a similar mechanism of action as itaconate by activating NRF2 and target enzymes in glycolysis [60]. DMF inhibits SARS-CoV-2 replication and replication of other viruses such as VACV, Zika virus, HSV1/2 in an interferon independent mechanism (see Table 1) [32]. DMF also suppresses inflammatory responses seen in severe COVID-19 by both liberating NRF2 and inhibiting the NF-κB signalling pathway by preventing p65 nuclear translocation. This has been shown to inhibit the transcription of inflammatory cytokines IFNB1, CXCL10, and CCL5 gene expression and upregulation of anti-inflammatory genes HMOX1 (see Figure 2) [32, 61]. Furthermore, activating NRF2 also blocks transmembrane protease serine 2 (TMPRSS2) expression, which is needed for ACE2-mediated cell penetration of SARS-CoV-2 via the spike protein [62, 63]. Given that DMF is an approved medication further research on whether it reduces severity of COVID19 is warranted.

## Conclusion

Studies into how SARS-CoV-2 affects intracellular metabolism during infection reveals interesting biochemical features, including increased glycolysis and dysregulated oxidative phosphorylation in innate immune cells. Preventing these metabolic changes in immune cells such as macrophages might yield new anti-viral and anti-inflammatory therapies. Itaconate might have potential antiviral and anti-inflammatory potential. The mounting of a robust innate immune response in the early phase of SARS-CoV-2 infection is important to reduce viral load. However, the ability to limit the viral-mediated metabolic reprogramming in host immune cells and other infected cells to reduce the excessive inflammatory response seen in the later phase of SARS-CoV-2 infection is an interesting prospect for the treatment of individuals who progress to severe COVID-19. The anti-viral and immunomodulatory properties of itaconate derivatives and DMF may give us two for the price of one; inhibition of viral replication and suppression of inflammation. Given these features, further studies with these metabolic modulators are well justified.

## Data Availability

Not applicable.

## Abbreviations

ACE2: Angiotensin-converting enzyme 2
ACOD1: Aconitate decarboxylase 1
CCL5: C-C Motif Chemokine Ligand 5
CXCL-10: C-X-C motif chemokine ligand 10
DMF: Dimethyl fumarate
GLUT-1: Glucose transporter 1
HCV: Hepatitis C
HIF1α: Hypoxia-inducible factor 1-alpha
HMOX1: Heme Oxygenase 1
HSV1: Simplex Virus-1
HSV2: Herpes Simplex Virus-2
IFNβ: Interferon beta
IL: Interleukin
IRF-3: Interferon regulatory factor 3
IRG1: Immune responsive gene 1
ISG15: Interferon stimulated gene 15
4-OI: 4-Octyl itaconate
LDH-A: Lactate dehydrogenase A
LPS: Lipopolysaccharides
Mtb: *Mycobacterium tuberculosis*
mTOR: Mechanistic target of rapamycin
mtROS: Mitochondrial ROS
NETs: Neutrophil extracellular traps
NF-κB: Nuclear factor kappa B
NLRP3: NOD-, LRR- and pyrin domain-containing protein 3
NLRs: NOD-like receptors
NqO1: NAD(P)H Quinone Dehydrogenase 1
NRF2: Nuclear factor-erythroid factor 2-related factor 2
NS5A: Non-Structural Protein 5A
OAS2: 2′-5′-Oligoadenylate Synthetase 2
PBMCs: Peripheral blood mononuclear cells
PFKFB3: 6-Phosphofructo-2-Kinase/Fructose-2,6-Biphosphatase 3
PKM2: Pyruvate kinase M2
PPAR: Peroxisome proliferator activated receptor
PPP: Pentose phosphate pathway
PRR: Pathogen recognition receptor
RAS: Rennin-angiotensin system
RET: Reverse electron transport
RIPK1: Receptor-interacting protein kinase-1
RIPK2: Receptor-interacting protein kinase-2
RSV: Respiratory syncytial virus
T2DM: Type 2 diabetes mellitus
TCA: Tricarboxylic acid cycle
TMPRSS2: Transmembrane protease serine 2
TNF: Tumour necrosis factor
VACV: Vaccinia virus
WNV: West Nile Virus
ZBP1: Z-form nucleic acid sensor
ZIKV: Zika virus
